# Implementing volunteer-navigation for older persons with advanced chronic illness (Nav-CARE): a knowledge to action study

**DOI:** 10.1186/s12904-020-00578-1

**Published:** 2020-05-22

**Authors:** Barbara Pesut, Wendy Duggleby, Grace Warner, Emily Kervin, Paxton Bruce, Elisabeth Antifeau, Brenda Hooper

**Affiliations:** 1 School of Nursing, University of British Columbia Okanagan, 1147 Research Road Arts 3rd Floor, Kelowna, BC V1V 1V7 Canada; 2 Faculty of Nursing University of Alberta, 3-141 ECHA 11405 87th Ave, Edmonton, Alberta T6G1C9 Canada; 3 Dalhousie University, P.O. Box 15000, Halifax, Nova Scotia B3H 4R2 Canada; 4 Mount Saint Vincent University, 166 Bedford Highway, Halifax, Nova Scotia B3M 2J6 Canada; 5 University of British Columbia Okanagan, 1147 Research Road. Arts 3rd Floor, Kelowna, BC V1V 1V7 Canada; 6 Regional Clinical Nurse Specialist, Palliative End-of-Life Care Services, Interior Health, c/o 2nd floor, 333 Victoria Street, Nelson, BC V1L 4K3 Canada

**Keywords:** (3–10) volunteers, Palliative, Public-health, Palliative approach, Compassionate community, Navigation, Hospice, Knowledge translation

## Abstract

**Background:**

Nav-CARE is a volunteer-led intervention designed to build upon strategic directions in palliative care: a palliative approach to care, a public health/compassionate community approach to care, and enhancing the capacity of volunteerism. Nav-CARE uses specially trained volunteers to provide lay navigation for older persons and family living at home with advanced chronic illness. The goal of this study was to better understand the implementation factors that influenced the utilization of Nav-CARE in eight diverse Canadian contexts.

**Methods:**

This was a Knowledge to Action study using the planned action cycle for Nav-CARE developed through previous studies. Participants were eight community-based hospice societies located in diverse geographic contexts and with diverse capacities. Implementation data was collected at baseline, midpoint, and endpoint using qualitative individual and group interviews. Field notes of all interactions with study sites were also used as part of the data set. Data was analyzed using qualitative descriptive techniques. The study received ethical approval from three university behavioural review boards. All participants provided written consent.

**Results:**

At baseline, stakeholders perceived Nav-CARE to be a good fit with the strategic directions of their organization by providing early palliative support, by facilitating outreach into the community and by changing the public perception of palliative care. The contextual factors that determined the ease with which Nav-CARE was implemented included the volunteer coordinator champion, organizational capacity and connection, the ability to successfully recruit older persons, and the adequacy of volunteer preparation and mentorship.

**Conclusions:**

This study highlighted the importance of community-based champions for the success of volunteer-led initiatives and the critical need for support and mentorship for both volunteers and those who lead them. Further, although the underutilization of hospice has been widely recognized, it is vital to recognize the limitations of their capacity. New initiatives such as Nav-CARE, which are designed to enhance their contributions to palliative care, need to be accompanied by adequate resources. Finally, this study illustrated the need to think carefully about the language and role of hospice societies as palliative care moves toward a public health approach to care.

## Background

Providing compassionate care for an aging population is a pressing social concern. Older persons who are at the highest risk for poor quality of life are those aging with multiple complex chronic illnesses [1]. As these persons transition from chronic illness toward end of life, they frequently experience challenges related to mobility impairments, sensory changes, and unrelieved symptoms [2–4]. In turn, these challenges can lead to dependence, social isolation, and poor quality of life [5]. Indeed, a recent multi-national study of seniors in 11 countries indicated that 1 in 5 Canadian seniors had experienced emotional distress that they had difficulty coping with in the last 2 years, and 17% reported feeling isolated some of the time to often [6].

To better support older persons living with advanced chronic illness, we developed a volunteer-led navigation intervention called Nav-CARE (Navigation—Connecting, Accessing, Resourcing, Engaging). Specially trained volunteers visit older persons living at home with advanced chronic illness to provide social support and facilitate connections to resources. Nav-CARE was designed to build upon four strategic directions for palliative care: (1) a palliative approach to care; (2) a public health/compassionate community approach to care; (3) the changing nature of volunteerism; and, (4) maximizing quality of life through navigation.

Although palliative care has made substantial progress over the past decades, there remain significant gaps. These gaps include the failure to identify individuals on a dying trajectory which results in late, or no, palliative care; a focus on palliative care in cancer to the exclusion of other life-limiting illnesses; and an emphasis on palliative care as a place rather than a philosophy of care which results in an overemphasis on specialized palliative care [7]. To redress these gaps, the concept of a palliative approach to care has emerged. A palliative approach to care is an early approach to care, for all those living with all life-limiting illnesses, which is delivered across healthcare contexts including both specialized care and primary generalist care [8–11]. It emphasizes teaching health care providers and other health partners to recognize and assist individuals and families living with advancing life-limiting illness who experience symptom burden, distress or quality of life issues with an appropriate plan of care.

A related approach to redressing palliative care gaps has been a public health approach to palliative care. Like a palliative approach, this approach focuses on early intervention; however, it does so by emphasizing and building upon the positive assets available through personal and social relationships [12–14]. Activities characteristic of this approach include public education, policy development, social marketing, and environmental changes [13]. From a public health approach, four sectors are essential for high quality palliative care: specialist palliative care, primary palliative care, personal social networks, and civic engagement [15]. This approach has been catalyzed through the compassionate community movement in which practice and policy coalitions are formed to promote the development of these four sectors [16, 17].

Hospice volunteers are an essential part of a robust palliative care system [18–20]. In the Canadian context, the volunteers are recruited, trained, and mentored through community-based hospice organizations. They provide services in the community, in free-standing hospices, and in institutional settings such as acute and long-term care. These volunteers have an important humanizing and relational role in care, a role that has been described as an intermediary role between healthcare professionals and family [21]. Further, the hospice organizations that support these volunteers perform an essential role in palliative policy and service development [21], and in educating communities about palliative and end of life care [22]. However, it is widely recognized that hospice volunteers are an underutilized resource [23, 24]. Despite the important role they perform in palliative care, patients and families are often referred to hospice either too late, or not at all, and hence, hospice volunteers are not used as much as they could be. In light of these contributions, further evidence is required about volunteer impact upon patient and family well-being [25].

Alongside these important historical contributions of hospice volunteers, it is important to note changing trends in volunteerism. In the Canadian context, volunteers in all sectors donate over two billion hours of service annually, with 47% of Canadians averaging 156 h each year. The contributions of volunteers to social cohesion, and the reciprocal well-being incurred by volunteers, are widely acknowledged [25, 26]. Although the overall contributions of volunteers in Canada have remained relatively stable, the type of work volunteers want to do is changing. Volunteers are looking for opportunities in which they can use the knowledge and skills they have developed during their lifetime in various careers [27]. As baby boomers retire from healthcare-related backgrounds, we can anticipate that many will want to use that experience within hospice palliative care. To capitalize on these new directions, we need to ensure that volunteer roles are meaningful for volunteers, build a strong evidentiary account of these volunteer contributions, and solve the perennial problem of the underutilization of hospice volunteers.

Navigation provides an intriguing opportunity for enhancing the contributions of hospice care volunteers. Professional, peer, and lay navigation models have been used widely within cancer care [28–31] and have been proposed as a way to reduce disparities in palliative care [32] such as those that might occur for palliative older persons living in rural areas [33]. Professional navigation models of care focus on navigating patients through healthcare services, whereas volunteer or lay models of navigation seek to solve quality of life issues that arise as a result of chronic and palliative illness experiences [34].

Nav-CARE is a volunteer navigational model that was designed to optimize a palliative approach to care, to provide new roles for hospice volunteers, and to support hospice’s role in building social cohesion and public engagement. As such, Nav-CARE is a community-based program that helps realize the ideal of a compassionate community approach to palliative care. Navigation is defined as:

*working in collaboration with patients, families, and communities to: a) negotiate the ‘best fit’ for the needs of persons, their families, and communities and resources; b) improve access to needed services and resources at the end of life (including death) and bereavement; and c) promote quality of life, foster independence, and facilitate community connections utilizing a culturally safe, palliative approach* ([34] p. 1).

Nav-CARE was developed and refined through several pilot studies [35, 36]. Building upon the pilot work, the goal of this study was to better understand the implementation factors that influenced the development of Nav-CARE in eight diverse Canadian contexts. In a related paper, we describe the outcomes of Nav-CARE [Authors in review].

## Methods

### Approach

**Fig. 1:**
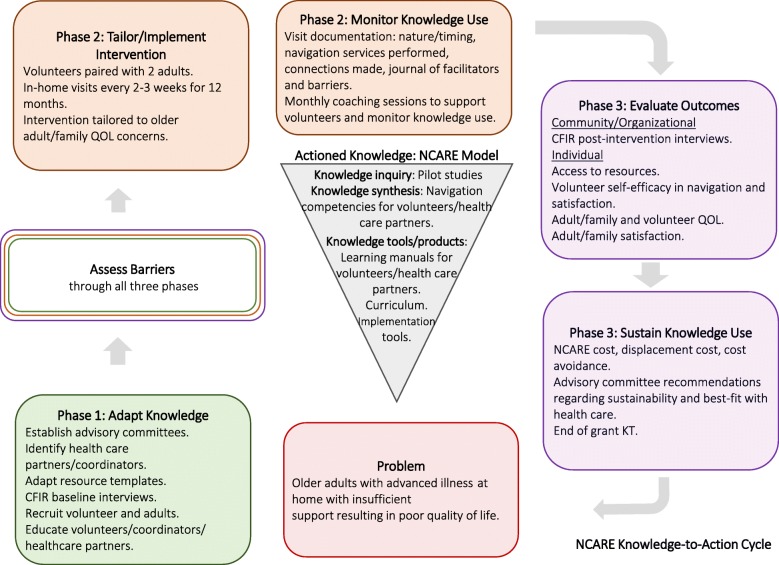
Implementing Nav-CARE using the Knowledge to Action Cycle. In this figure the Knowledge to Action Cycle is adapted to show the actioned knowledge of Nav-CARE, the problem that Nav-CARE addresses, and the details of Nav-CARE’s adaptation, implementation, and evaluation across participating communities

This was a knowledge to action (KTA) project using the KTA framework developed by Graham and colleagues [37]. The KTA framework has two components: (1) knowledge creation and (2) a planned action cycle that uses the knowledge created and adapts it to each context while assessing barriers, monitoring knowledge use, evaluating outcomes, and sustaining knowledge use. Figure 1 is an adaptation of the KTA framework for this project.

### Knowledge creation

The Nav-CARE knowledge, which was used in the planned action cycle described in this paper, was developed through previous studies that entailed constructing the conceptual and theoretical foundations for older adult navigation [33]; creating, testing, and refining curriculum for volunteer navigators [38]; and incremental pilots to test feasibility and acceptability [35, 36]. From these studies, a Nav-CARE implementation toolkit was created that includes an implementation manual for organizations, a volunteer training manual, and comprehensive curricular materials so that organizations could train volunteers independently.

Nav-CARE was designed to be implemented through community-based volunteer societies and is implemented in the following manner. Societies set up an Advisory Committee for Nav-CARE implementation and appoint a volunteer coordinator to oversee Nav-CARE. The coordinator is responsible for recruiting and supporting volunteers; recruiting and screening older persons and family; matching older persons and volunteers; and raising the visibility of Nav-CARE in the local community. Volunteers are recruited, trained, and matched with older persons. The majority of volunteers recruited were already hospice volunteers, those who were not had equivalent volunteer experience. After volunteers are trained, they visit older persons and family in the home every 2 to 3 weeks. Although friendly visiting is an important aspect of the intervention, volunteers are also trained in navigational activities such as assisting with decision-making and advance care planning; locating resources and assisting with accessing those resources; and supporting client engagement with desired activities. Visits continue until death, assuming that both volunteers and older persons want to continue the relationship.

### Planned action cycle

The planned action cycle for Nav-CARE was 12 or 18 months depending upon the study site. Three sites focused specifically on a cancer population with a 12-month intervention; five sites focused on older persons living with advanced illnesses, including cancer, with an 18-month intervention. The project was conducted in the three phases of the KTA process: *Phase 1***:**Adapt knowledge to local context. The goals of this phase were to develop community-specific advisory committees; compile baseline data from each community to aid in understanding the community context; recruit volunteers and persons; and deliver navigation education. The process entailed conducting informational meetings for stakeholders to inform them of the Nav-CARE intervention, gaining commitment from the organization for participation, planning for implementation in cooperation with key stakeholders, collecting baseline data, and recruiting volunteers, older persons, and family. Coordinators at study sites were responsible for recruiting volunteers and older persons. Volunteers and volunteer coordinators, and in some cases healthcare providers, underwent 2 days of training by the research team.

*Phase 2***:**Tailor intervention and monitor knowledge use. The goals of this phase were to implement the volunteer navigation intervention and collect process data. Ongoing mentorship was provided by an experienced nurse navigator through monthly 2-h teleconferences. Regular teleconferences were also held for volunteer coordinators for the purposes of sharing implementation experiences.

*Phase 3***:**Evaluate outcomes and sustain knowledge use**.** The goals of this phase were to describe the contextual factors that influenced Nav-CARE implementation, evaluate outcomes at the community, organizational, and individual level [Authors, in review], sustain the intervention, and conduct end of grant KT.

### Participants

Nav-CARE was implemented in eight community-based hospice societies across diverse urban and rural contexts. Study sites were located in British Columbia, Alberta, and Nova Scotia. Only one site was urban (population of greater than 100,000). Two sites were small urban and three sites were rural. Characteristics of these communities can be found in Table 1.

**Table 1:** Characteristics of Communities in in which Hospice Societies Resided

Site	Population	Distance to Urban	In-patient hospice beds
1	9000	60	No
2	48,000	NA	Yes
3	10,000	350	No
4	150,000	NA	Yes
5	5000	60	No
6	12,000	90	No
7	19,000	400	No
8	10,000	400	No

Participating hospice societies had differing capacities and services. Two of the eight societies had in-patient hospice beds. Seven of the eight societies had an existing home visiting program and one of these societies had an established home visiting program for seniors who were not palliative. One small urban society only had volunteers working on an in-patient hospice; in that situation Nav-CARE was a way to revive a pre-existing home visiting program. The degree to which these societies had formal affiliations with healthcare varied. Only one of the six societies was fully integrated with a health region (i.e., supported primarily through healthcare dollars). The remaining seven received varying levels of support, but a large part of their funding came from private donations.

Participants across the eight intervention sites included older persons (*n* = 49), family (*n* = 38), and volunteers (*n* = 38). In addition, 55 stakeholders (beyond those who participated as volunteers and older persons) from both hospice and healthcare settings provided data about their perceptions and experiences of the Nav-CARE intervention.

To help offset some of the costs of participating in this project, each society received $200 per month which was matched through in-kind donations supplied by the organization. Volunteers received $40 per month to help offset some of the costs associated with participating in the research (e.g., filling out forms to supply research data and participating in interviews).

### Data collection and analysis

Implementation data was collected through semi-structured qualitative individual and group interviews with stakeholders (e.g., hospice society directors and board members, volunteer coordinators, volunteers, healthcare providers, Nav-CARE advisory committee members) at baseline, midpoint, and conclusion of the intervention (See Table 2). Baseline interviews were conducted with those in leadership in hospice and healthcare and focused on the need for Nav-CARE in the community, how Nav-CARE fit with the organization’s strategic plan, and what factors they anticipated would affect Nav-CARE implementation. Midpoint and endpoint interviews were conducted with those stakeholders who had intimate knowledge of the process of Nav-CARE implementation and development (e.g., hospice leaders, volunteers, coordinators, advisory members). This meant that fewer stakeholders participated in the endpoint interviews than in the baseline interviews. These interviews focused on understanding facilitators, barriers, and recommendations for improvement. Implementation issues arising throughout the action cycle were documented as field notes. Data were also collected from email and phone correspondence with coordinators and other leaders, regular coordinator teleconferences, and through volunteer mentoring sessions conducted via teleconference. Further, volunteers kept visit logs in which they recorded visit statistics (e.g., length and frequency of visits), navigation activities completed, and successes and challenges encountered (see Tables 3 & 4).

**Table 2:** Individuals who Participated in Evaluation Interviews

Baseline		Midpoint		Endpoint	
Individual (n)	Focus Groups (n)	Individual (n)	Focus Groups (n)	Individual (n)	Focus Groups (n)
NA Not part of study protocol	8 focus groups with 55 stakeholders	H = 1 VC = 9 V = 4 *P* = 9 F = 2	NA Not part of study protocol	VC = 4 V = 22 *P* = 12 F = 10	5 focus groups with 16 stakeholders

Interviews conducted with the following in total: *H* Hospice Stakeholders (*n* = 55), *VC* Volunteer Coordinators (*n* = 9), *V* Volunteers (*n* = 27), *P* Adult Participants (*n* = 21), *F* Family (*n* = 9)

**Table 3:** Quality of Life Focus for Older Persons, Family, and Navigators

Older Person Quality of Life Concerns	Family Quality of Life Concerns	Navigator Quality of Life Focus
1. Pain levels and other symptoms (E.g. SOB, confusion, dizziness etc.) 2. Changes to eyesight, hearing, and taste 3. Energy levels 4. Concerns for family and pets 5. Financial matters 6. Relocation/housing 7. Companion and/or assistance with groceries, shopping 8. Treatment side effects 9. Healthcare professionals 10. Own or others’ accomplishments 11. Faith/spirituality 12. Outlook on life and death 13. Uncertainty of illness trajectory 14. Special occasions/Visits/Trips 15. Home healthcare products/devices 16. Level of independence 17. Home and vehicle maintenance 18. Meals and food choice 19. Homecare 20. Advance care planning/future planning 21. Issues with elimination (e.g. incontinence, bowel irregularity) 22. Weather 23. Ability to participate in recreation/hobbies 24. Spousal or family illness 25. Physical therapies and complementary therapies 26. Relationships/Friendship 27. Phone and TV connection 28. Mobility 29. Transportation 30. Direct care needs 31. Mental health 32. Caregiver respite 33. Meaningful connection and reflection	1. Respite time 2. Caregiver burnout 3. Supporting client’s changing needs 4. Keeping realistic expectations 5. Interpersonal conflict 6. Too many people involved in client’s care 7. Emotional impact of caregiving 8. Social isolation 9. Future planning 10. Financial concerns 11. Concern about client’s symptoms/illness 12. Managing or accessing help with household chores and errands 13. Concern for client’s care arrangements 14. Balancing own health concerns with caregiving 15. Anticipatory grief 16. Finding appropriate resources/answers from HCPs 17. Transportation 18. Self-care	1. General social support 2. Access to home medical equipment and supplies 3. Financial assistance 4. Transportation 5. Easy access to appropriate healthcare personnel and medications 6. Open communication/planning for end of life 7. Companion or other solution for outings or errands 8. Ability to participate in hobbies 9. Assistance with maintaining home 10. Better symptom control 11. Support for/knowledge of client’s specific condition 13. Ability to “get out and about” 14. Mental health support 15. Means to communicate (e.g. phone line) 16. Assistance with meals 17. Emergency/short notice contact 18. Meaningful connection/source of meaning-making.

**Table 4:** Barriers, Successes, Learnings as Documented by Volunteers

Barriers	Successes	Learnings
➢ Family and client tensions ➢ Lack of a perceived need for navigation ➢ Difficulties contacting clients or arranging visits ➢ Finding resources, particularly financial ➢ Client symptoms that influenced engagement with navigation ➢ Uncertainty of the illness progression ➢ Emotional impact of long-term relationships with clients. ➢ Managing boundaries	➢ Enhanced client motivation and independence ➢ Ease of navigator/client relationship (friendship development) ➢ Facilitating connections with client’s family ➢ Open and honest communication, including discussions around bad news, illness trajectories, and advance care planning ➢ Legacy conversations (i.e., documenting client’s life story) ➢ Connecting clients with community resources	➢ Client’s support needs will fluctuate alongside their illness ➢ It is not easy to find resources/answers ➢ Clear boundaries are integral to the success of a navigator/client relationship ➢ Frequent and consistent visits and/or check-ins make the navigation process “richer” ➢ Families can identify issues that clients may not ➢ The client/navigator relationship is highly relational and personally impactful ➢ The navigator role is often one of the “friendly visitor”; listening and being present are key

Qualitative data were uploaded to NVivo (a qualitative data analysis software) and analyzed using qualitative description [39]. A code book was developed based on the study questions and was used to guide analysis. Initial interview transcripts were coded by two investigators to ensure coding integrity and a thematic account of the data was constructed. Quantitative data were entered into SPSS for analysis, cleaned, and analyzed using descriptive statistics.

The study was approved by three university research ethics boards. Data was amalgamated across study sites to protect participant confidentiality.

## Results

We will begin by presenting the baseline organizational data which were collected to better understand Nav-CARE’s fit with the organization and anticipated facilitators and barriers. We will then describe the data collected at the mid- and end-points of the intervention that addressed contextual factors that influenced Nav-CARE outcomes. To aid in interpreting the data, hospice stakeholders (H); volunteer coordinators (VC); volunteers (V); older adult participants (P); and family (F) will be identified for each quote. However, to protect confidentiality no identifying numbers are assigned that could link individuals back to their community. Further, while we have used the terminology of older person in this paper, volunteers in this study often referred to them as “clients” in their quotes.

### Baseline organizational context

Baseline data collection was undertaken to better understand the community organizations and to capture their perspectives about the fit between Nav-CARE and their organization. These included fit with their long-term vision, a compassionate community approach, and anticipated resources and barriers.

#### Fit with long term vision

Stakeholders (e.g., hospice board members and staff) who were interviewed perceived Nav-CARE to fit well with the long-range vision of their society by broadening the scope of palliative care upstream in the health system, facilitating hospice outreach into the community, and changing the public perception of hospice care. In other words, if Nav-CARE was successful, then hospice societies would no longer be associated only with care of the imminently dying. “*We’re trying to move that care way upstream and work with people much earlier in their disease trajectory*.” (H) The program was thought to mesh well with other providers of home care (e.g., home care nurses and health care assistants) designed to keep older persons living at home independently. Stakeholders believed that volunteers would play an important and complementary role that was not provided by the public healthcare system.

#### A compassionate community approach

Many stakeholders spoke about Nav-CARE as an opportunity to more broadly de-medicalize palliative care, to bring palliative care in line with a compassionate community approach, and to facilitate hospices’ community education and capacity-building role.

“*We are more than just the four walls. I am envisioning that the program will be so well known in the community that someone will think ‘oh so and so is having difficulty getting around and life is becoming tough’ and they will think of this program just like they think of hospice when someone is dying.”* (H)

Most hospice societies had community outreach as a key element of their strategic plan. Stakeholders further expected the program to avert palliative crises in their communities by having volunteers act as a surveillance system in which problem solving would occur proactively. “*Most of what we see is reactive – not proactive. You see people trending down but nothing happens until they crash*.” (H) Stakeholders expressed a critical need for Nav-CARE and were able to envision individuals in the community who would be a good fit for the program.

#### Anticipated resources and barriers

When asked what resources would be required to implement Nav-CARE, participants identified strong community and healthcare partnerships and community awareness. When asked to identify the factors that would most likely influence the success of Nav-CARE, participants cited the quality of the volunteers, community awareness of the program, and the credibility it generated by providing a valuable service. “*In the long run we will have to take a careful look at how many volunteers we are going to need and what strain that puts on us, because they are not something you can go out and buy*.” (H) Conversely, potential barriers to successful implementation included the stigma associated with hospice; the inability to meet the demand for services if the program became well known; and the privacy and independence of rural individuals that might influence recruitment. “*One barrier could be the client’s reluctance to have strangers in the home. My father needed all kinds of services but he just couldn’t adjust to having people in his home*.” (H).

### Contextual factors that influenced Nav-CARE outcomes

The most significant factors affecting Nav-CARE program implementation were attributes of the volunteer coordinator; organizational capacity and connectedness; the ability to recruit older persons; and volunteer preparation and mentorship.

#### Coordinator champion

The volunteer coordinator who acted as a champion for Nav-CARE in the community was the single most important factor in successful implementation. The study team worked closely with each volunteer coordinator through extensive email and phone conversations. It became clear over the intervention that the coordinator’s role in taking ownership of implementation and seeing it through was the most significant predictor of success. Nav-CARE coordinators required a unique set of leadership and mentorship skills. They could only perform their role well when they had sufficient support from their organization, local healthcare services (e.g., physicians), and the advisory committee. The coordinator position could be time-intensive, particularly if there were client recruitment challenges. For example, field notes taken of coordinator teleconferences indicated that these individuals were expending significant amounts of time trying to identify older persons for their volunteers.

##### Leadership

Coordinators’ leadership skills were the central strength and dynamism that maintained the network of participants and stakeholders. The visibility role, in which coordinators were tasked with raising awareness of Nav-CARE in the local community, required that they be comfortable connecting with various stakeholders through presentations and networking. Field notes taken throughout the intervention period illustrated the many ways in which coordinators connected with organizations and persons serving seniors in their community (e.g., seniors centers, healthcare providers, town councils). In that sense, their role was a very public one and required a degree of confidence in interacting with multiple stakeholders. Further, to carry out this role they had to be comfortable with the aims of Nav-CARE and the population it sought to serve. This message was easily lost when coordinators changed through the study period.

##### Mentorship

Coordinators were also required to be present for, and to support, volunteers. Volunteers suggested that the willingness and capacity of the volunteer coordinator to support them was one of the most important aspects of their involvement. “*You can’t find a better coordinator to keep us all in the loop and who we call if we don’t quite know how to handle something.”* (V) The ability to effectively match navigators and older persons in terms of personality and approach was mentioned as a key skill of the volunteer coordinator. A family member commented on this phenomenon. “*I think her ability to match the absolute perfect person with the volunteer. It was almost like she knew her volunteers.”* (F) Another navigator commented on the important role of the coordinator in helping them to navigate unanticipated client situations or rapid decline. “*If there are red flags, coordinators need to be proactive around reassessing. She’s [coordinator] been around the block here, she’s seen a lot of people, she was on it when her [the client’s] illness flared up.”* (V).

##### Support from colleagues

Even as coordinators provided support for the volunteers, they too required support in this new role. The Nav-CARE research facilitator met regularly with coordinators by phone. The regular teleconferences held for coordinators were viewed as particularly helpful. “*I knew that that if anything did come up, I had a weekly phone call scheduled with the research facilitator. That was absolutely fantastic.”* (VC) This regular contact with the research team and with volunteer coordinators across the country was essential for support and for sharing challenges and solutions, particularly as Nav-CARE was a new program.

##### Support from healthcare providers

Nav-CARE was designed to be implemented in partnership with healthcare providers. The implementation toolkit encouraged organizations to develop at least one healthcare partner as part of the program. Coordinators concurred that to sustain the program, they needed a strong active relationship with at least one healthcare partner. A coordinator made the following suggestion. “*I think having a contact with home care, somebody that you can always compare your notes with and touch base with.”* (VC).

Unfortunately, coordinators in some communities had different experiences regarding their healthcare partner and when these linkages did not work, the lack of connections negatively impacted program outcomes and made the coordinator’s role more difficult.

*Well, it goes back to the very beginning with just trying to find the person in the Health Authority who could connect and begin to try and collaborate with … for referrals and to see where the program would best fit and how we could work together. There was just resistance, almost animosity, even when I would approach nurses, the palliative care nurses, social workers, even our healthcare partner. At first there seemed to be a lot of excitement, but then it was just pull … with the palliative care program it sort of seemed like they just right away were, this isn’t a program for us, go see this person. And I’d go see that person and it would be, go see this person, and it kind of went on like that … So, there just didn’t seem to be even an interest to learn about it. It sort of was more than resistance.* (VC)

These quotes demonstrate how obstacles can occur when health professionals in a community perceive Nav-CARE to be duplicating their own professional roles. Although healthcare partners received orientation to the Nav-CARE volunteer role through presentations early in the project, this knowledge did not extend to other healthcare personnel who became involved with volunteers through the intervention period.

##### Support from advisory committee

Establishing an Advisory Committee to support the volunteer coordinator was an important part of implementation. Advisory committees were meant to provide practical guidance, to help raise the public profile of Nav-CARE, and to assist with recruiting. In several communities, coordinators did not experience their advisory committee to be as helpful as expected. In some communities, advisory members did not attend meetings or assist with client recruitment. A coordinator expressed her frustration: “*I really expected a little more. I guess I really didn’t know what to expect. So, it has been somewhat disappointing. To say the least.”* (VC) When coordinators changed during the project, there were no pre-existing relationships with Advisory Committee members. “*Our advisory committee could be more supportive. Because it was initiated and started before I came on, I don’t have the relationship with those people. So, I haven’t used the advisory committee the way that I think it could be used and maybe better utilized.” (VC)* In some communities, coordinators perceived the selection and make-up of the advisory committee to be a barrier, whereas in other communities, it was more related to the lack of communication between the coordinator and advisory committee. A coordinator reflected on future changes and how committee make-up might improve the program outcomes. “*I would maybe look at getting more of the arms of healthcare, like the physiotherapists, maybe acupuncturists … I’m not too sure. Even just somebody that does hands-on and works with these clients maybe?”* (VC) Despite disappointments in some communities, the potential of advisory members was also articulated. In one community, the coordinator planned to find ways to better engage her advisory committee. “*I think the advisory committee is really key. And I think there’s some work that could be done around how to get buy-in and to define the role of the advisory committee a little bit better and how to stay engaged with the committee.”* (VC).

##### Sufficient time

Finally, the time allocated for coordination influenced how coordinators experienced their role. Once a program was established, some coordinators felt that little time would be required to keep it running smoothly. For example, a coordinator said, “*I think once it’s up and running, maybe a guesstimate would be 4 or 5 hours if you’re busy that month?”* (VC) However, in other communities, coordinators expressed concern about workload and the number of navigators they could effectively supervise if the program successfully expanded. “*Having four volunteers is more than enough for me. If I had, you know, six, eight, ten on this particular program I probably couldn’t manage.”* (VC) This particular coordinator felt this pressure because she perceived navigators to need a lot of support, which drew on her time. Pacing the workload required flexibility and the ability to respond to unforeseen developments. A coordinator described the uneven nature of the role’s responsibilities and the need to adapt, especially related to the intake of new older persons and time commitments. “*I found when we all of a sudden had seven referrals, and going out and doing the initial contacts, I found that was quite a time demand, that many all at once.”* (VC).

In summary, the coordinator role was pivotal to the success of Nav-CARE. A coordinator who had strong leadership skills, mentoring abilities, and community connections was critical to program implementation. Good relationships with a healthcare partner and advisory committee members could potentially support the coordinator in their role; however, these relationships were developed with varying degrees of success.

#### Organizational capacity and connections

Nav-CARE was implemented through non-profit, community-based societies, and as such, was influenced by factors that influenced their organizational capacities. For example, the partnerships, key leaders, and degree of organizational disruption all influenced the capacity to implement and sustain Nav-CARE.

##### Partnerships

Organizations that had strong and pre-existing partnerships with healthcare providers experienced the most straightforward implementation of Nav-CARE. However, Nav-CARE also required new partnerships because hospice societies were typically partnered with healthcare providers delivering late palliative care, while Nav-CARE was being delivered to an early palliative population. This gap meant that the organizations needed to develop stronger connections with the primary care system. For example, this study site developed what they described as the perfect connection to healthcare through a primary care system partner: “*because she has the role of Integrated Care Coordinator, she facilitates the necessary care between physicians and clients and other resources. So, she’s definitely the go-between for these clients.”* (VC) But, for a number of study sites, this connection did not happen. In some sites the volunteer coordinators did not have the time to make the connections, in other sites healthcare providers did not respond to these attempts at partnership. In contrast, rural community sites did not experience this same difficulty as primary and palliative care were provided by the same healthcare providers. In these communities, the partnerships required to access this upstream population remained the same; however, this did not necessarily mean that these healthcare providers would realize the contributions of hospice to this new population, and hence, refer patients.

##### Key leaders

As part of an integrated knowledge translation approach, study sites were involved in planning the research project and were partners on the grant funding. However, during this implementation period, five out of the six study sites had changes in leadership. New leaders appeared to be not as well-informed about Nav-CARE nor did they have the same degree of buy-in as those leaders originally involved in the project. New leaders needed time to get established in their role and simply did not have the time or energy to champion Nav-CARE as a new program. Conversely, the two sites that experienced the fewest personnel changes had the most success implementing Nav-CARE in a timely fashion.

##### Organizational disruption

In addition to the changes in leadership three of the six organizations also experienced significant organizational disruptions during the study period that entailed changes to both resources and personnel. For example, one study site lost their facility, one study site was engaged in a highly public controversy, and one experienced a significant new direction that generated conflict within the Board. Another site with limited resources was attempting to implement two new programs concurrently. For those hospice societies with in-patient hospice beds, the legalization of medical assistance in dying created significant workload and organizational disruption. Overall, these disruptions influenced the resources available for implementing a new program.

##### Sustainability

The ability to sustain Nav-CARE beyond the study period was largely determined by organizational capacity. At the conclusion of the research period, all sites continued to care for the registered older persons and family. However, four of the sites stated they were not going to sustain the program over time. One site reversed this decision after 1 year. Another site situated Nav-CARE in different community-based organization in which it was re-initiated and sustained. Reasons for discontinuing the program were largely related to resources, both in terms of finances for a volunteer coordinator and concerns that Nav-CARE volunteers would deplete their existing volunteer resources. However, 1 year after study conclusion, six of the eight hospice societies have maintained their Nav-CARE program and have recruited and trained additional volunteers.

#### Recruitment

Older person recruitment posed the most significant barrier to implementing the Nav-CARE program. At the beginning of the intervention, all sites had concerns about not having the capacity to handle the number of older persons that could potentially come forward. These concerns persisted even though the research team had explained to participating communities that recruitment was one of the most challenging issues in the early pilot studies. This concern caused communities to take an overly cautious approach to recruiting in the early part of the intervention. For example, organizations steered away from any recruitment strategies that they felt could overwhelm their capacity. While study sites were encouraged to use a combination of strategies for client recruitment including public advertising for self-referral and healthcare referrals, some sites were concerned that making the service public would set up an expectation they could not fulfill. For example, data from field notes indicated that one volunteer coordinator stopped seeking to partner with physicians when one physician suggested he could have 100 patients for her right away. By the time organizations realized that it might be difficult to recruit, several months had already elapsed since the volunteer training. The research team worked individually with sites to strategize client recruitment; however, many strategies were unsuccessful. Time to first client recruitment varied from 3 weeks to 9 months across communities. Three communities were never fully recruited (i.e., a 2 to 1 ratio of older persons to volunteer navigators).

##### Recruitment strategies

Participating community sites used various combinations of the following recruitment strategies: public advertising (e.g., media interview, press releases, brochures), community talks (e.g., presentations to societies serving seniors), and focused communication with healthcare providers (e.g., presenting at team meetings, letters to physicians, meetings with strategic partners). Participants were surprised by the difficulty they encountered with client recruitment because they perceived a great need for the program, as indicated in the baseline focus groups. A coordinator reflected. “*You know we reached out to so many different places. And I was very surprised how long it took us to get clients and how there wasn’t the kind of uptake that we thought was going to be there.”* (VC) A healthcare partner in a community that experienced difficulty identifying older persons said, *“I think that piece is a really big and a fascinating element to this research and trying to figure out”, “Okay, if it worked so well on these other communities, what was the struggle for our community? What’s different about our community?”* Participants identified two factors they felt influenced client recruitment: public perceptions of palliative care and professional gatekeeping.

##### Public perceptions of palliative care

The public perception of hospice societies influenced the ability to recruit older persons who did not see themselves as ‘*hospice clients*.’ For example, coordinators spoke of losing eligible older persons as soon as they called the organization and heard the word hospice over the telephone. In that way, hospice societies were faced with the challenge of reshaping their identity. Participants described how the perception of hospice and palliative care is associated with imminent death, as opposed to living and dying well. For example, an older person declared s/he would definitely recommend the program but added that, *“people don’t want to think about hospice”* (P). This participant went on to explain that he knew elderly neighbours who would benefit from the Nav-CARE program but they did not want to know about it because it was associated with hospice. A navigator described the same issue working with older persons and her experience using the term “palliative care.” “*I think it would be a lot more helpful to them, you know, as things go along, to get into palliative care early, but as soon as you mention that word ‘palliative care’ they think, oh God, I’m going to die.”* (V) A navigator who lost two older persons because they withdrew from the program accounted for this by noting the use of the term “palliative” in materials they read. “*Well, at first I thought, did I do something wrong? And then I realized that in the packaging that they had read, some of the wording in it was palliative. And they felt that they were not ready for palliative care. They wanted to focus on living and doing the best they could with her condition, rather than preparing for her leaving.”* (V) Similarly, another navigator asserted the term “hospice” was a barrier to the program. *“A lot of people hear the word hospice and they’re afraid because they think it’s all about dying. And so, it needs to be sort of reframed so that people think of it as a support and not about death.”* (V) In summary, hospice societies were chosen as community partners because of their historical success in supporting older persons to travel the palliative journey. However, the stereotypes of hospice as only serving clients at end-of-life made it difficult to recruit older persons.

##### Professional gatekeeping

What volunteers described as gatekeeping was another barrier to recruitment. Many participants felt that healthcare providers in their community would be the primary means of client recruitment. However, they soon discovered that, for a variety of reasons, healthcare providers were not referring older persons. One participant suggested that selecting the right healthcare partner early on in the intervention was key to success.

*Had we picked somebody, a healthcare partner that was actually more invested or integrated into the health authority, then that might have been a little bit easier. But it didn't seem like any of the physicians or anybody in the palliative care world out here were really open to even thinking about giving this as an option, and I know that was a really big frustration for us.* (VC)

A navigator in a different community described the same barrier regarding health professionals’ resistance, this time attributing it to a lack of appreciation of the volunteer contributions.

*In some communities, the professional support people, like homecare nurses, social workers, can be very possessive and territorial. They don't feel that a volunteer is appropriate, that it's almost as if the volunteer is taking over their job, which is really a shame because in most cases they work really, really well together.* (V21)

Although what appeared to be gatekeeping was a barrier across several communities, a healthcare partner went on to explain that in her community the healthcare system had an early referral to palliative care and for this reason, Nav-CARE was perhaps perceived as *“duplicating that service.”* Nonetheless, like other communities, she also identified systematic barriers to recruitment among health professionals. *“It’s important to get in with the family doctors, the seniors’ clinic doctors, because I think they are a huge obstacle to get referrals and to get people connected.”* (H) A coordinator who fully supported the program, but who had experienced difficulty recruiting lamented that the “*disappointing thing about it was the lack of referral and the disinterest in promoting the program from the medical profession, doctors, and home care.”* (VC).

There were, however, communities who recruited effortlessly, but even in these instances, buy-in from the medical community was lacking. In one smaller community that quickly met their client quota, the coordinator explained that they had excellent support from their healthcare partner.

*We utilized doctors’ offices, but we didn’t have a lot of buy-in. Our healthcare partner, our social worker was excellent. She referred 3 of the present clients that we have. And I obtained the others through basically word of mouth about what we were doing. So, I don’t know what the actual strategic plan would be to actually recruit [laughter] … just putting posters up didn’t seem to do it. I would like to get more buy-in from the physicians, personally.* (VC)

Low recruitment was a barrier in itself, but it also caused low morale for navigators eager to help older persons. A navigator expressed her disappointment, noting the lack of support from healthcare professionals. “*I was disappointed that there were not more clients. We didn’t have the support of the medical staff, of the doctors in town, or the people out there, like homecare … and we didn’t have the support so I never did get a second client.”* (V).

Interestingly, participants commented on a sense of resistance that they perceived to be widespread among the healthcare professionals in their community. Anecdotally, healthcare providers spoke of the challenges they encountered in having sufficient time to provide the type of care that Nav-CARE volunteers provided. They spoke of a time passed when they too had the luxury of extended conversations with their older persons. As one healthcare provider said, “*that is what my job used to look like*.” Part of the resistance, therefore, may be the perception that volunteers are being used to replace paid healthcare staff.

##### Recommendations for enhancing recruitment

Stakeholders had a number of recommendations for improving recruitment of older persons in the community. The number one recommendation was better integration and engagement with the healthcare system. The role of an active healthcare partner was cited as important to sustaining the program. Creating better linkages within the healthcare system, especially among family doctors, to obtain referrals was also seen as critical. One coordinator felt that a formal arrangement with home care organizations represented the best possibility for identifying potential older persons.

Another common suggestion was to improve advertising and marketing. Many participants, navigators and older persons recommended that the program be more strongly promoted in the community. One navigator simply said, *“advertise, advertise, advertise to ensure the local community is fully aware of the program.”* (V) Many navigators suggested more advertising in local newspapers; one navigator suggested face-to-face presentations might be more effective than print, such as an information seminar at the public library. (V) A coordinator talked about the need to advertise continuously through several mediums to ensure the program’s sustainability. “*Flyers - that would go into everybody’s mailbox. We have a paper that comes out weekly, having an ad run for a month. Things like that. Posters that are made up and are placed in all these facilities, on a regular basis, like every 3 months or something. I think, having it in people’s faces continuously.”* (VC) Public perceptions of hospice that influenced communities’ abilities to recruit older persons led to volunteers and other stakeholders considering other, non-hospice related, partnerships such as senior’s centers or meal delivery organizations. A coordinator who experienced difficulty with recruitment in her community talked about the need to re-emphasize the role of the volunteer in palliative care in general. She pointed out how the concept of the volunteer is absent in training, education, and medical discussions about palliative care. She made the following big picture observation.

*Why are we not talking about the role of a volunteer in palliative care? Because I think that would … that would make it easier. It would give us an open door to begin discussions with healthcare … And so, not just us as the [Hospice Association] going in and doing a presentation or the university going in and doing a presentation about NAV-Care. Even a bit farther upstream, where do we fit in the role of the volunteer in quality palliative care?*(VC)

Most of these suggestions were part of what had been recommended to study sites as part of Nav-CARE implementation. However, materials supplied to the organizations for recruiting were not user friendly; rather, they reflected the ethical requirements of a research study. As a result, the research team hired a company to produce public and healthcare provider Nav-CARE awareness materials (e.g., posters, brochures, prescription pads) that also met study requirements. These materials were made available to all study sites and became part of the implementation toolkit. Further, as suggested above, part of the reason for lack of public awareness was the resources (both time and money) that were required to do this type of visibility-raising work.

#### Volunteer preparation and mentorship

All study sites were able to easily recruit qualified volunteers to take the two-day Nav-CARE training. This training covered six modules: introduction to the volunteer role; assessing client and family quality of life; advocating for clients and families; facilitating community connections; supporting access to services and resources; and promoting active engagement. Evaluation of the Nav-CARE education immediately post training indicated a high degree of satisfaction. The learning climate was positive and participants felt well prepared for their new role. Role-play, skits, and discussions were the most valuable learning strategies. Communication strategies and self-care were the most valuable content for many participants.

Participants were also asked about the effectiveness of the training in the qualitative interviews near the conclusion of the intervention. Participants suggested that improvements were needed in three areas: adapting the education to volunteers’ prior knowledge; being clearer about volunteer scope; and, providing ongoing education and mentoring after the initial workshop.

##### Adapting education to prior knowledge

Although all of these participants were hospice volunteers, some had extensive background in palliative care as healthcare providers whereas others did not. These differences were reflected in how prepared participants felt for the volunteer role. For example, the following participant did not have a healthcare background. “*We’re not social workers, we’re not nurses, so when working with such vulnerable people we need a little bit more education.”* (V). One coordinator suggested that it was essential for volunteers to have a healthcare background. “*I think just taking volunteers from wherever, is not adequate. They do need to have some experience behind them.”* (VC).

##### Knowing volunteer scope

Part of the debate around adequate preparation arose from misunderstandings about the volunteer role in navigation. “*In the training, have clearer guidelines as to what is okay and what is not okay to do as a volunteer navigator.”* (V) This concern around boundaries was echoed by a volunteer coordinator.

*“I came out of the training feeling like the volunteers were going to be asked to give advice on things and that’s something I wasn’t really comfortable with. Where is that line between a volunteer visitor and a volunteer navigator? What exactly is being asked of these volunteers and is it something that they’re comfortable with?”* (VC)

This lack of clarity around the role was evident when two navigators objected to the task of developing a thorough knowledge of community resources, despite this knowledge being central to the volunteer navigator role. *“Do I need to know all this stuff in the community? It can just waste a lot of time phoning around to all these things”* (V).

##### Ongoing education and mentorship

Data collected at mid-point that addressed the confusion around boundaries and the need for further education and mentorship lead to adaptations in Nav-CARE implementation. The monthly coaching calls for navigators were revised to include more structured educational content around topics such as boundaries, self-care, and medical assistance in dying. Navigators suggested that this adaptation was important in increasing their confidence in their new role.

In addition to education around their role, navigators also expressed a need for emotional support in light of their long-term relationships with older persons. “*The other thing that is really, really, really, really important is support for the navigators - I don’t want to say counseling, but someone to be able to go and talk to because I know a number of the people who have lost clients and it has affected them personally.”* (V) Those sites that gathered together as a group to debrief in person were the most effective in providing this type of emotional support. However, one navigator suggested that this was better done one-on-one rather than in a group meeting. “*Maybe I would feel comfortable with just another navigator, rather than in a group and just say, ‘Do you want to just meet for coffee sometime and just go over stuff?”* (V).

In summary, although the workshop was evaluated positively, there was a need to further determine the background expertise that volunteers should have prior to becoming a volunteer, to clarify the role and boundaries of volunteers, and to provide ongoing education and emotional support. This support is best provided within the organization where volunteers can debrief with other volunteers.

## Discussion

The purpose of this study was to better understand the implementation factors that influenced the development of Nav-CARE within eight diverse Canadian contexts using the KTA framework. These factors included organizational capacity, the coordinator champion, client recruitment, and volunteer training and mentorship. One of the primary limitations of the study was the nature of self-report obtained through interviews, meetings, and conversations with study sites. We learned of additional factors that undoubtedly influenced Nav-CARE development but may not have been captured within the dataset. These were sensitive topics around interpersonal relationships and the public profile of organizations. Another limitation was that Nav-CARE depended largely upon continuity in organizational leadership. However, this was something that rested entirely within the control of organizational partners. In this discussion we will further explore the relationship of volunteer satisfaction to support; the importance of champions to community-based initiatives; the delicate balance between hospice underutilization and organizational capacity; and, the challenges of realizing a palliative, public health approach in hospice.

### Volunteer satisfaction and support

The relationship of volunteer satisfaction to appropriate education and support has been well-documented in the literature [40, 41]. Volunteers desire ongoing education and a chance to be connected with others who understand the difficulties of their role. Nav-CARE made those needs even more urgent. Volunteers were taking on an entirely new role that, although it had familiar elements of friendly visiting, also had new competencies unique to navigation. Volunteers received training in the workshop about the role and its boundaries; however, once they were working with older persons they found that there was much to discover and work out on their own. The qualitative data collected from the volunteer visit logs and from the qualitative interviews indicated that these volunteers, in large part, took on this new role and adapted to it successfully. Providing ongoing education for the volunteers in partnership with the mentorship sessions was an important adaptation. In future workshops, it will be important to prepare volunteers by normalizing this initial role confusion, at least until the role of a Nav-CARE volunteer develops a more robust culture of its own. An important adaptation will be to incorporate more information in the implementation manual around Nav-CARE volunteer roles in relation to professions, those providing paid manual work, and unpaid caregiving. Further, it will be important to reconsider whether having a background in healthcare should be a requirement for the role. Although some participants suggested that this should be the case, this might further complicate the boundaries between professional and volunteer involvement within healthcare teams [42]. The gatekeeping that occurred in this study, and the perception that volunteers were being used to replace professionals might become more pronounced.

### Importance of community-based champions

The primary factor that determined how well the program developed was the volunteer coordinator. The importance of community-based champions in palliative program development has been well documented by Kelley in her studies on rural palliative care capacity building [43–45]. The coordinator champions in this study were typically pre-existing volunteer coordinators within the hospice organization. As such, within the context of Nav-CARE they took on familiar functions of recruiting and overseeing volunteers. However, the role in which Nav-CARE coordinators were required to promote a new program through community networking may have been new to them. It is typically the Board and Executive Director of hospice societies who perform key roles in palliative policy and service development [22, 46]. Further, although most hospice societies aspire to a more community-engaged role, for many this vision remains largely unrealized [47].

The coordinator skills required by the Nav-CARE roles of community developer and mentor of volunteers were substantially different, albeit both required highly developed relational skills. Mentoring volunteers required one on one supportive relational skills whereas community development required public relational skills. Volunteer coordinators in this study engaged their communities to varying degrees—some establishing multiple community connections, others few. The coordinators who were able to easily integrate Nav-CARE into the community were those who already had long standing relationships with influential stakeholders. Based on this data, the volunteer implementation manual will be revised to reflect the competencies required of the volunteer coordinator; dividing the role between two people with complementary skill sets may be a better option. However, altering the role in this way has resource implications and may further dilute the important role of that central Nav-CARE champion.

### Hospice underutilization and capacity

This study illuminated important aspects of hospice and hospice volunteer underutilization that have been documented in the literature [23, 24]. Typically, this underutilization is attributed to a misunderstanding of the vital role that volunteers can play in improving the quality of life of patients and families [48]. In this study, that misunderstanding was compounded by the assumption that volunteers were now taking on roles that healthcare providers used to have time to perform. This belief, in turn, led to what participants described as gatekeeping which had an adverse effect on recruiting older persons. Similar gatekeeping issues affecting the recruitment of older persons living with advanced illness have been described in the literature [49]. Healthcare providers are protective of the well-being of their patients and concerned about important roles being given to volunteers for economic reasons. In future research it would be important to explore these issues more fully with healthcare stakeholders.

Furthermore, there was another important dynamic related to underutilization—that of limited organizational capacity. Pre-implementation data indicated that these study sites were familiar with the needs of this population and felt they could make an important contribution to meeting those needs, although they were also very cautious about overwhelming their capacities. Indeed, all sites were initially concerned about the numbers of older persons that might request their services. It is important to note that in trying to optimize their contributions to palliative care, sites were implementing other new programs at the same time, most notably hospice day care programs.

Our findings raise a complex question: if we believe that volunteers are essential to high quality palliative care [18–20], then how can we better support these volunteer organizations to ensure their optimal contributions? Without adequate support, the line between offering meaningful services and becoming overwhelmed by the need becomes precarious. The paradox is that volunteers make contributions that healthcare providers are not paid to do, and so the role is thought to fall outside of paid healthcare services. However, if policy documents indicate that volunteers are truly integral members of palliative teams [19, 20], then funding for the structures that support those volunteers is essential. Such payment has the potential to enhance the legitimacy of volunteer organizations, perhaps paving the way for better integration with paid services. Participants in this study identified that integration as a critical factor to success of building and sustaining Nav-CARE.

### A palliative, public-health approach in hospice

Establishing how to adapt hospice societies to the changing face of palliative care has become a central question arising from this study. If palliative care is indeed everyone’s business (the public health approach) [12–14] and if it needs to happen early on (the palliative approach) [8–11], how might the longstanding and revered culture of hospice need to adapt? Older persons in this study were immediately discouraged from taking advantage of a service offered by an organization traditionally thought to care for the actively dying. Further, in keeping with a palliative approach to care, much of care is now being assigned to interdisciplinary chronic illness management teams. The strong relationships developed between hospice societies and palliative care practitioners may no longer give hospice access to the upstream palliative population. Organizations in this study were feeling the effects of these changes. There is an urgent need to re-envision the role of hospice, and how to language its services, in light of these developments. In this study, hospice boards were already actively considering their long-term vision in consideration of these changes. Indeed, the dedication of these sites to Nav-CARE sustainability suggested that they see this new role for hospice as a top priority. However, these issues also need to be tackled beyond the local level through a policy approach.

## Conclusion

Hospice organizations are well positioned to support important palliative care policy directions of a palliative approach and public health approach to care. Volunteer navigators show significant promise for improving the quality of life of older persons living at home with advanced chronic illness. In the context of the Nav-CARE intervention, it was essential for organizations to have an organizational champion, healthcare connections, and organizational capacity to be successful. These essential components were largely realized through the social capital that these organizations had already developed within their communities. In our current work, we are scaling out Nav-CARE to additional sites across Canada to build an evidence-base of its potential reach and impact.

## Data Availability

The datasets generated and/or analyzed during the current study are not publicly available for privacy reasons but are available from the corresponding author on reasonable request.

## Abbreviations

KTA: Knowledge to Action
H: Hospice stakeholder
VC: Volunteer coordinator
V: Volunteer
P: Older adult participant
F: Family
